# Cervical Disc Arthroplasty (CDA) versus Anterior Cervical Discectomy and Fusion (ACDF) for Two-Level Cervical Disc Degenerative Disease: An Updated Systematic Review and Meta-Analysis

**DOI:** 10.3390/jcm13113203

**Published:** 2024-05-29

**Authors:** Chiu-Ming Chen, Jui-Jung Yang, Chia-Chun Wu

**Affiliations:** Department of Orthopaedics, Tri-Service General Hospital, National Defense Medical Center, Taipei City 11490, Taiwan

**Keywords:** degenerative disc disease (DDD), cervical disc arthroplasty (CDA), anterior cervical discectomy and fusion (ACDF), efficacy, two levels

## Abstract

**Background**: Anterior cervical discectomy and fusion (ACDF) and cervical disc arthroplasty (CDA) are both considered to be efficacious surgical procedures for treating cervical spondylosis in patients with or without compression myelopathy. This updated systematic review and meta-analysis aimed to compare the outcomes of these procedures for the treatment of cervical degenerative disc disease (DDD) at two contiguous levels. **Methods**: The PubMed, EMBASE, and Cochrane CENTRAL databases were searched up to 1 May 2023. Studies comparing the outcomes between CDA and ACDF in patients with two-level cervical DDD were eligible for inclusion. Primary outcomes were surgical success rates and secondary surgery rates. Secondary outcomes were scores on the Neck Disability Index (NDI) and Visual Analogue Scale (VAS) for neck and arm pain, as well as the Japanese Orthopaedic Association (JOA) score for the severity of cervical compression myelopathy and complication rates. **Results**: In total, eight studies (two RCTs, four retrospective studies, and two prospective studies) with a total of 1155 patients (CDA: 598; ACDF: 557) were included. Pooled results revealed that CDA was associated with a significantly higher overall success rate (OR, 2.710, 95% CI: 1.949–3.770) and lower secondary surgery rate (OR, 0.254, 95% CI: 0.169–0.382) compared to ACDF. In addition, complication rates were significantly lower in the CDA group than in the ACDF group (OR, 0.548, 95% CI: 0.326 to 0.919). CDA was also associated with significantly greater improvements in neck pain VAS than ACDF. No significant differences were found in improvements in the arm VAS, NDI, and JOA scores between the two procedures. **Conclusions**: CDA may provide better postoperative outcomes for surgical success, secondary surgery, pain reduction, and postoperative complications than ACDF for treating patients with two-level cervical DDD.

## 1. Introduction

Cervical degenerative disc disease (DDD) leads to a rise in neck and arm pain, as well as radiculopathy and myelopathy, which significantly affect patients’ quality of life [1]. The treatment approach for cervical DDD focuses on alleviating discomfort, enhancing functional abilities, and reducing the recurrence and duration of symptoms. Initially, nonoperative conservative interventions are typically employed, but in cases where nonoperative measures are ineffective, surgical intervention may be warranted [2]. Cervical DDD that is refractory to nonoperative treatment is a common condition encountered by spine surgeons today, and it places a significant cost burden on the healthcare system [3].

Anterior cervical discectomy and fusion (ACDF) and cervical disc arthroplasty (CDA) are commonly performed procedures considered to be efficacious for the treatment of cervical spondylosis in patients with or without compression myelopathy [4]. ACDF has long been considered the gold standard approach for managing symptomatic cervical DDD [5]. Nonetheless, it has been associated with various postoperative problems, including cervical spine range of motion restrictions and the accelerated degeneration of neighboring segments, known as adjacent segment disease (ASD) [6]. Within ten years of the initial procedure, it has been shown that ACDF causes ASD in up to 25% of cases, necessitating secondary surgery [7,8,9].

CDA has garnered increasing attention in recent years owing to its perceived advantages. The annual number of cervical disc replacements performed is witnessing steady incremental annual increases of approximately 17% [10]. Many comparative studies, as well as systematic reviews, have extensively evaluated the short- and long-term outcomes of ACDF and CDA for single-level cervical DDD [11,12,13,14,15]. In general, CDA has been shown to produce better results than ACDF, including higher overall success rates, lower pain scores for both arm and neck, and lower reoperation rates for discs replaced at adjacent levels. Additionally, CDA provides significant advantages over ACDF by maintaining mobility capability at the injured levels and maximizing the restoration of the biomechanical qualities of an intact cervical spine [11,12,13,14,15].

A recent meta-analysis compared the short- and long-term results of CDA with ACDF; however, despite two-level surgeries being performed in some of the studies included, this meta-analysis did not distinguish between single-level and two-level surgeries and analyze them separately [16]. The most recent evaluation focused on two-level degenerative disc disease only included research up to 2015, necessitating an updated meta-analysis to address this gap in the literature [17]. Consequently, the goal of this systematic review and meta-analysis was to examine the current published literature to compare outcomes of CDA versus ACDF for two-level cervical DDD.

## 2. Methods

### 2.1. Search Strategy

This systematic review and meta-analysis was conducted in accordance with the Preferred Reporting Items for Systematic Reviews and Meta-Analyses (PRISMA) guidelines [18]. A literature search was conducted through major public databases (i.e., PubMed, EMBASE, and Cochrane CENTRAL) using the keywords “anterior cervical discectomy and fusion”, “ACDF”, and ”two-level”, combined with Boolean operators and using Medical Subject Heading (MeSH) terms where appropriate for studies published up to 1 May 2023. As an example, the search string used for PubMed was as follows: (arthroplasty OR replacement) AND (anterior cervical discectomy and fusion OR ACDF) AND (two-level). In addition, the reference lists of included studies were hand-searched to identify other potentially relevant studies.

### 2.2. Selection Criteria

This review was performed in accordance with the PICOS criteria (participants, intervention, comparison, outcomes, study design). Eligible studies were those investigating patients with two-level cervical DDD (P) and surgically treated with CDA or ACDF (I&C), which reported at least one of the outcomes (O) of interest, including the overall success rate, secondary surgery rate, Neck Disability Index (NDI), neck or arm pain visual analog scale (VAS), Japanese Orthopedic Association (JOA) score, and postoperative complications. Only published RCT, two-arm retrospective, and prospective studies were considered (S).

Review articles, letters, commentaries, editorials, proceeding research, meeting abstracts, case reports, and personal communications were excluded. Studies with a language other than English were also excluded. The eligibility of studies identified through the above search and selection strategy was confirmed by two independent reviewers, and a third reviewer was consulted where there was uncertainty regarding eligibility.

### 2.3. Main Outcome Measures and Data Extraction

The primary outcomes of interest were the overall success rate and secondary surgery rate. The secondary outcomes were NDI, neck pain VAS, arm pain VAS, JOA scores and complication rates after surgery.

From these eligible studies and when available, the following information was extracted: name of first author, year of publication, study design, study country, number of patients undergoing CDA and ACDF, patients’ mean age, sex (male %), mean follow-up time, and main outcomes of interest.

### 2.4. Ethics Statement

This systematic review and meta-analysis of published studies neither required nor used raw patient data or private information; therefore, the approval of the protocol from the hospital’s institutional review board (IRB) and informed consent from study subjects were waived.

### 2.5. Quality Assessment

To assess the quality of the included studies, we utilized the Cochrane risk of bias (ROB) 1.0 tool for RCTs and the ROBINS-I tool (“Risk Of Bias In Non-randomised Studies-of Interventions”) for non-RCTs. The Cochrane ROB tool assesses the risk of bias using the following different criteria: selection bias (i.e., random sequence generation and allocation concealment), performance bias (i.e., blinding of participants and personnel), detection bias (i.e., blinding of outcome assessment), attrition bias (i.e., incomplete outcome), reporting bias (i.e., selective outcome reporting), and the inclusion of an intention-to-treat analysis [19]. Additionally, the ROBINS-I tool assesses the risk of bias using seven different criteria: bias due to confounding, bias arising from the measurement of the exposure, bias in the selection of participants for the study, bias due to post-exposure interventions, bias arising from the measurement of the outcome, bias in the selection of the reported result, and the overall risk of bias [20]. A quality assessment was performed by two independent reviewers, and a third reviewer was consulted for any uncertainties.

### 2.6. Statistical Analysis

Odds ratios (ORs) with 95% confidence intervals (CIs) were calculated for dichotomous outcomes, while standardized mean difference with 95% CIs were calculated for continuous outcomes. A χ^2^ test of homogeneity was performed, and heterogeneity among the studies was evaluated using the Cochran Q and I^2^ statistics. Heterogeneity determined using the I^2^ statistic was defined as follows: 0% to 24%, no heterogeneity; 25% to 49%, moderate heterogeneity; 50% to 74%, high heterogeneity; and 75% to 100%, substantial heterogeneity. When a smaller number of studies were included in the meta-analysis, the statistical power of the heterogeneity test was low [21]. If the power of the heterogeneity test was insufficient, a random effect model was used [22]. The pooled effect was calculated and displayed as an OR with a 95% CI or standardized mean difference, and a two-sided *p*-value < 0.05 was considered statistically significant. Publication bias was assessed using funnel plots. A sensitivity analysis was conducted using the leave-one-out approach in which a meta-analysis was performed with each study removed in turn. All analyses were performed using Comprehensive Meta-Analysis statistical software, version 2.0 (Biostat Inc., Englewood, NJ, USA).

## 3. Results

### 3.1. Search Results

**Figure 1:**
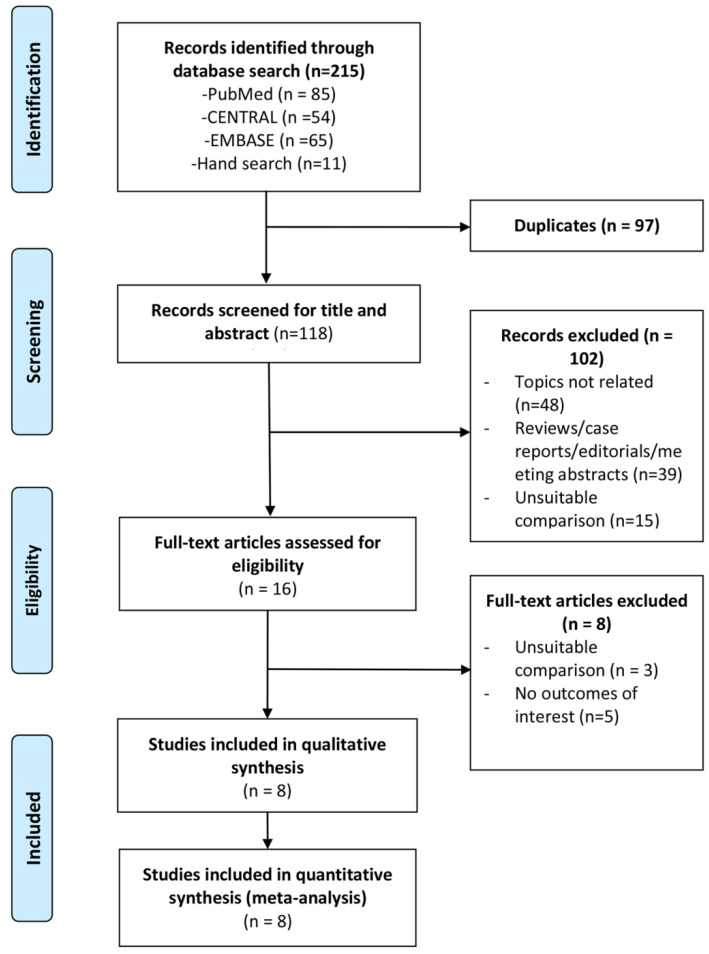
PRISMA flow diagram of study selection. The number of search hits are shown, corresponding to each step of the systematic literature search, qualitative review, and quantitative analysis. The basis for search hit exclusions are described.

The electronic search and study selection process is shown in Figure 1. After excluding duplicates, the search yielded a total of 118 unique citations, for which titles and abstracts were screened. A total of 16 full-text articles were assessed for eligibility, and 8 were excluded for not reporting outcomes of interest or having unsuitable comparisons. Finally, eight studies (two RCTs, four retrospective studies, and two prospective studies) were included in the systematic review and meta-analysis [23,24,25,26,27,28,29,30].

### 3.2. Characteristics of Included Studies and Baseline Patient Demographics

Table 1 summarizes the characteristics of the included studies and patients. The eight studies enrolled a total of 1155 patients with two-level cervical DDD. The patients were classified into a CDA group (n = 598) and an ACDF group (n = 557). The mean age was 46.8 years for CDA patients and 51.1 years for ACDF patients. Patients were followed from 13 months to 10 years.

### 3.3. Meta-Analysis of Overall Success Rate and Secondary Surgery Rate

**Figure 2:**
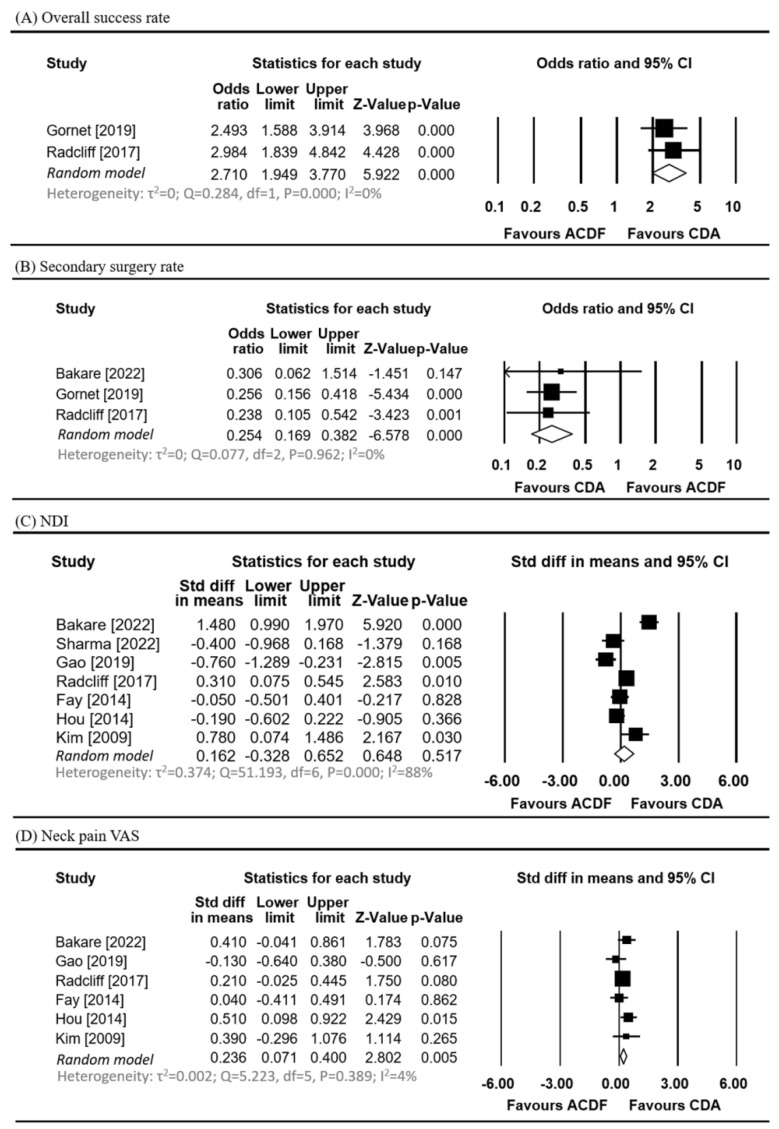

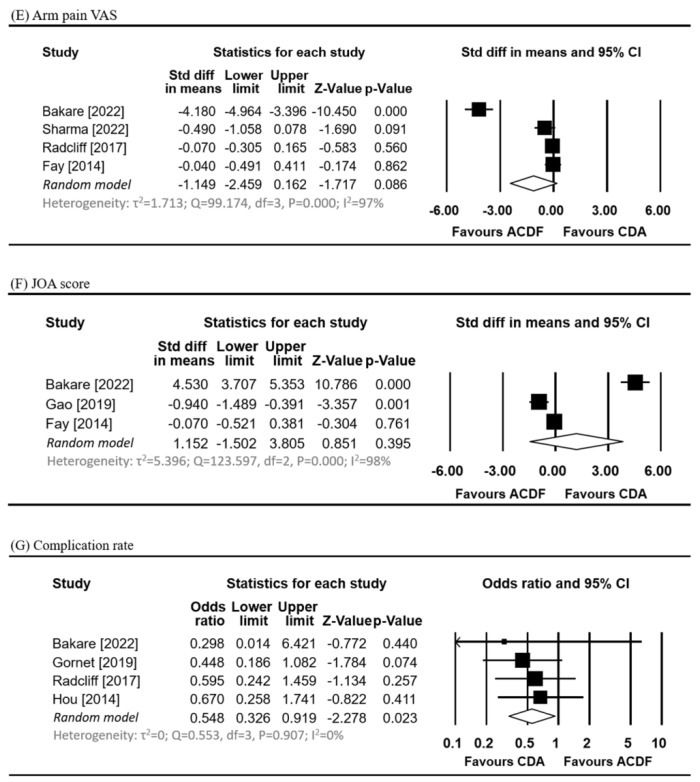
Meta-analysis of the (**A**) overall success rate [26,27], (**B**) secondary surgery rate [23,26,27], (**C**) NDI [23,24,25,27,28,29,30], (**D**) neck pain VAS [23,25,27,28,29,30], (**E**) arm pain VAS [23,24,27,28], (**F**) JOA score [23,25,28], and (**G**) complication rate [23,26,27,29].

Figure 2A,B show the results of the meta-analysis for the overall success rate and secondary surgery rate between CDA and ACDF. Two studies reported an overall success rate [26,27]. Heterogeneity was not observed (Q statistic = 0.284, I^2^ = 0%, *p* = 0.000). A significantly higher overall success rate was observed in the CDA group compared to that in the ACDF group (OR, 2.710, 95% CI: 1.949 to 3.770) (Figure 2A).

Three studies reported secondary surgery rates [23,26,27]. Heterogeneity was not observed across the studies (Q statistic = 0.077, I^2^ = 0%, *p* = 0.962). A significantly lower secondary surgery rate was observed in the CDA group compared that in the ACDF group (OR, 0.254, 95% CI: 0.169 to 0.382) (Figure 2B).

### 3.4. Meta-Analysis of Secondary Outcomes

Figure 2C–F show the results of the meta-analysis for changes from the baseline in the NDI, neck pain VAS, arm pain VAS, and JOA scores after CDA and ACDF.

Six studies reported changes in neck pain VAS [23,25,27,28,29,30]. Among these, a greater improvement in neck pain VAS was observed in the CDA group over the ACDF group (standardized mean difference: 0.236, 95% CI: 0.071 to 0.400, *p* = 0.005, I^2^ = 4%).

Seven studies reported NDI changes [23,24,25,27,28,29,30], four studies reported changes in arm pain VAS [23,24,27,28], and three studies reported changes in the JOA score [23,25,28]. The results of the meta-analyses showed no significant differences in changes in the NDI, arm pain VAS, and JOA scores between the two procedures (all *p* > 0.05).

Figure 2G shows the meta-analysis results for complication rates between CDA and ACDF. Four studies reported complication rates [23,26,27,29]. Among these, the CDA group had a lower complication rate (OR, 0.548, 95% CI: 0.326 to 0.919, *p* = 0.023, I^2^ = 0%) than the ACDF group.

### 3.5. Sensitivity Analysis and Publication Bias

**Figure 3:**
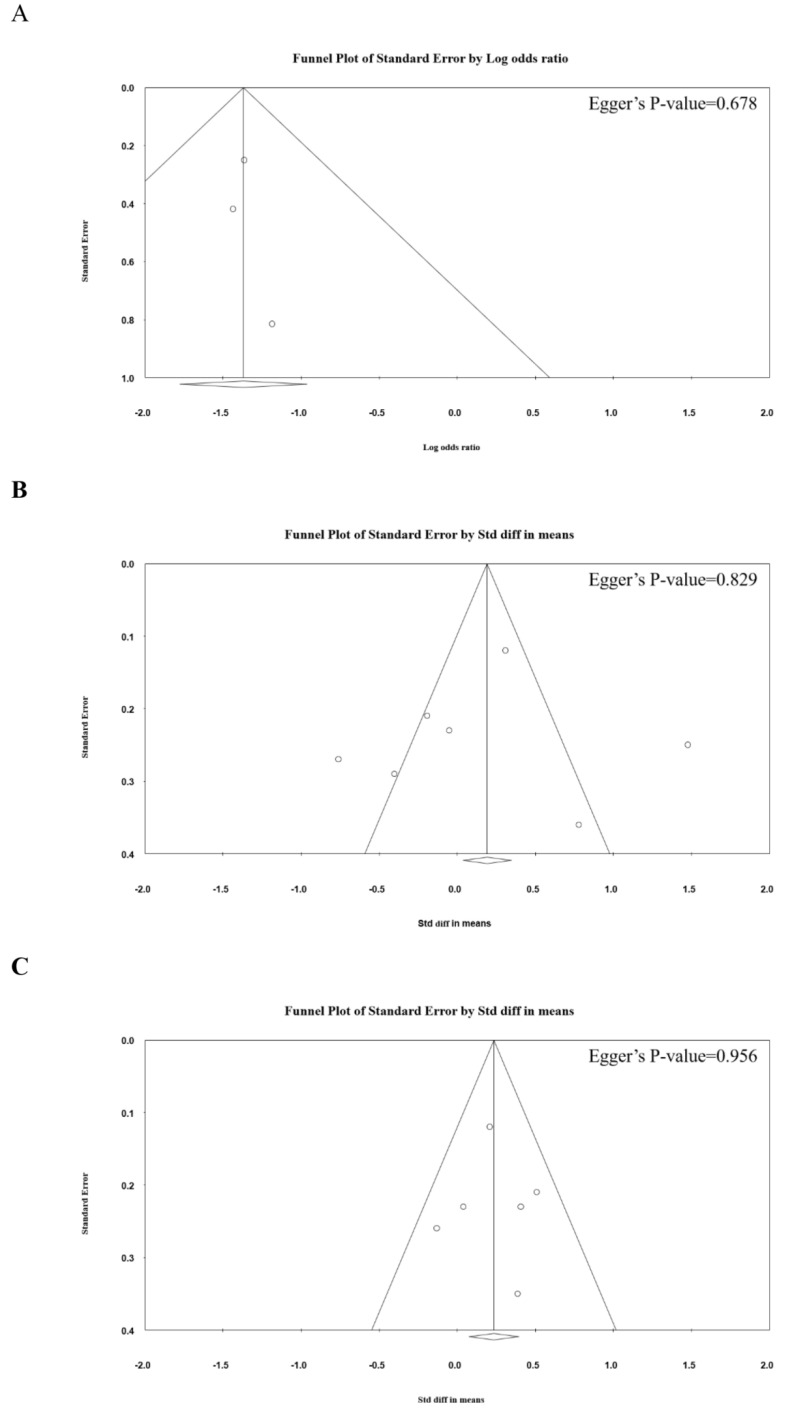

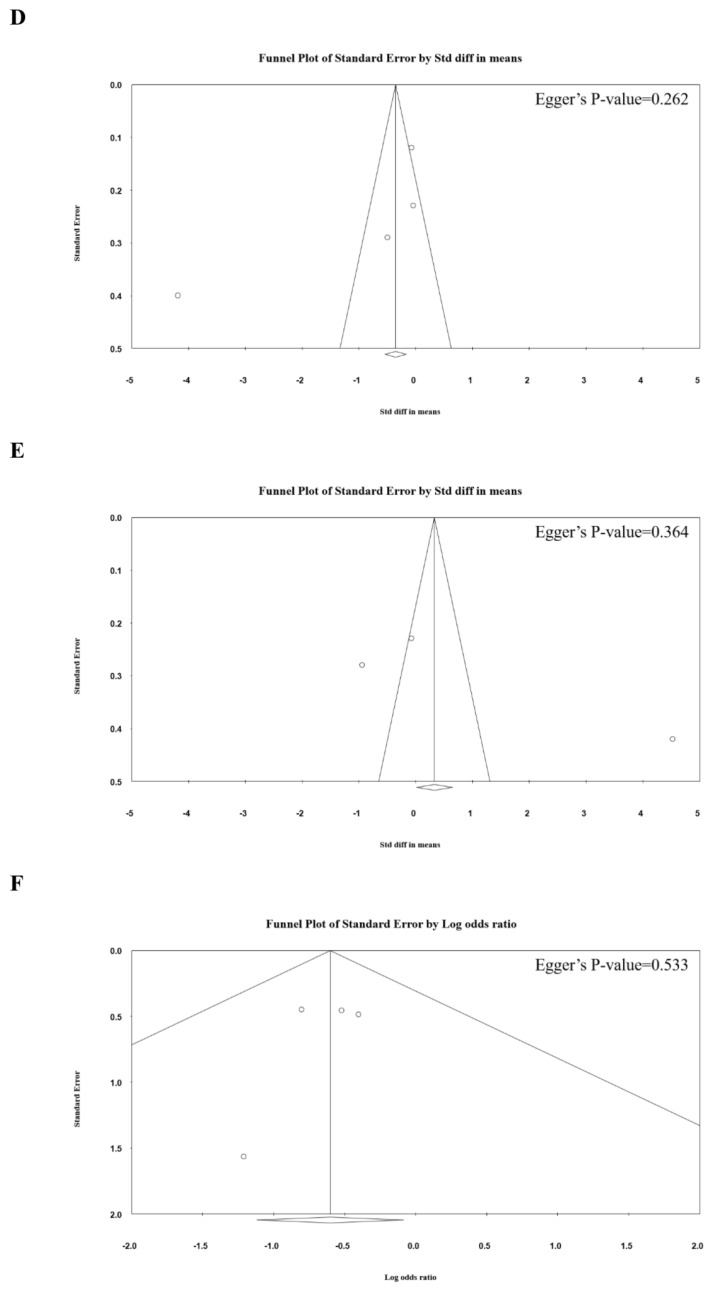
Funnel plots for the verification of publication bias in the present meta-analysis. Egger’s test was utilized to verify the presence of publication bias in the meta-analysis of the (**A**) secondary surgery rate; (**B**) NDI; (**C**) neck pain VAS; (**D**) arm pain VAS; (**E**) JOA score; and (**F**) complication rate.

Publication bias analysis results are shown in Figure 3A–F. Egger’s regression test suggested that there was no evidence of publication bias in the outcomes assessed (all *p* values > 0.05).

Sensitivity analysis results are shown in Table 2. The direction and magnitude of the pooled estimates for the overall success rate, secondary surgery rate, NDI, neck pain VAS, arm pain VAS, and JOA score did not vary markedly with the removal of any one study, indicating that the meta-analysis had good reliability and the data were not overly influenced by any given study. For complication rates, the difference between groups became not significant after removing the study of Gorent et al. (*p* = 0.129); however, there was no change in the magnitude or direction of complication rates after removing all other studies. Overall, the sensitivity analyses indicate that none of the studies overly influenced the pooled estimates, indicating that the results of the meta-analysis are robust.

### 3.6. Risk of Bias

Two RCTs and six retrospective studies underwent a thorough risk of bias evaluation. The overall risk of bias of the included studies was considered to be low. The detailed results of the risk of bias assessment for each study are documented in Supplementary Figures S1 and S2.

## 4. Discussion

The present updated systematic review and meta-analysis used the most recent studies to summarize the results of CDA vs. ACDF for treating two-level cervical DDD. The meta-analysis revealed that CDA has significantly higher overall success rates and lower secondary surgery rates over those of ACDF. CDA outcomes resulted in significantly greater improvement in neck pain VAS than ACDF, while improvements in the arm VAS, NDI, and JOA scores appear to be comparable between the two procedures. CDA is also associated with a significantly lower risk of complications than ACDF. The findings of this meta-analysis for the outcomes of CDA vs. ACDF suggest that CDA may be a more efficacious procedure than ACDF for treating patients with two-level cervical DDD.

Our study presents a unique perspective on the outcomes of two-level DDD surgeries, comparing CDA and ACDF. An earlier meta-analysis conducted by Gao et al. in 2013 revealed that ACDF was associated with shorter operative times and less blood loss compared with CDA [31]. While we did not specifically report on operative times and blood loss, our results somewhat differ from those of Gao et al., demonstrating higher overall success rates and reduced complication risks with CDA. Several reasons might explain these differences. Firstly, unlike Gao et al., who did not specify the number of levels treated, our analysis was strictly limited to two-level diseases. Additionally, all included studies in Gao et al. were published before 2011, whereas half of the studies we included were conducted after 2019. This time gap suggests that advancements in surgical techniques and improvements in clinical practices over the years could account for the variations in outcomes.

The overall success rates of CDA in the present meta-analyses were significantly higher than those shown for ACDF at two levels. A study included concluded that CDA and ACDF were both viable options for treating two-level DDD, with postoperative alignment changes demonstrated as equal between the two procedures [23]. Specifically, however, in a clinical trial and an earlier meta-analysis, CDA at either one or two levels was shown to be superior to ACDF in patient-reported outcomes, the range of motion of the cervical spine, and reductions in ASD [15,32]. Nevertheless, authors have cautioned that patient selection for these procedures must consider the clinical and functional aspects of each patients’ disease structurally, including sagittal alignment, which is the equilibrium between the cranium, spine, and pelvis, and urged surgeons to focus on patients who will benefit most from preserving mobility [23]. Differences in patient selection in that study included performing ACDF for those with severe ankylosis and loss of disc height, aiming to improve disc height and restore lordosis; while CDA was performed for those with disc-related disease and without rigidity or significant loss in disc height, aiming to restore healthy joint function, preserve mobility, and maintain lordosis.

CDA at two-level DDD was previously thought to be insufficient to produce significant advantages in sagittal alignment over ACDF [23]. An earlier review of 15 studies also found CDA to be non-inferior but also not superior to ACDF in short- and long-term outcomes [33]. In contrast, more recent studies contributing to our findings have reported the superiority of CDA for viable long-term outcomes, including maintaining the normal disc height, preserving segmental motion, and avoiding the side effects of fusion associated with ACDF, as well as reducing complications attributed to anterior cervical plating and cervical immobilization [25,26,27].

Further, the present meta-analysis showed that CDA had lower secondary surgery rates than ACDF at two levels. Regrettably, our analysis did not extend to the specific causes of secondary surgery—whether they stemmed from surgical-related complications, implant-related issues, or ASD—due to a lack of sufficient data and limited follow-up duration. ASD is a known postoperative complication of ACDF, with an incidence of 70% among patients with cervical two-level DDD [16]. It develops because of the increase in intradiscal pressure and segmental motion at levels adjacent to the fusion site. An additional surgery is often needed when ASD develops after procedures have been performed for cervical DDD initially [34]. Not specifically focused on two-level procedures, a meta-analysis that compared ACDF with artificial cervical disc replacement found that disc replacement was as effective as ACDF but reduced the development of possible complications, including ASD, hence reducing the associated secondary surgeries [16]. Performing CDA for two-level cervical DDD is noted for preserving mobility at the index levels without increasing adverse effects, which would help to reduce the need for re-operation later [28]. Nevertheless, that report still found that two-level arthroplasty and ACDF had similar outcomes up to 39 months postoperatively. Other reports included in this meta-analysis have shown that the incidence of secondary surgery, or re-operation, was all higher during different durations (short, medium, long) of postoperative follow-up for ACDF [25]. However, explanations for this trend vary between studies.

In this review, CDA was also associated with a significantly lower risk of complications than ACDF, which logically reduces secondary surgeries. Unfortunately, the data do not allow us to distinguish between implant or surgery-related complications, which warrants further investigations. Another important consideration is that patients with two-level cervical spondylosis may have more advanced degeneration, and consequently, implanted discs in these cases may be more subject to adverse events. In the literature, other complications associated with CDA or ACDF include wound infection, prevertebral or epidural hematoma, vertebral artery injury, cerebrospinal fluid leak, and the migration of the prosthesis; dysphagia and hoarseness may also occur with either CDA or ACDF [35]. One of the studies included in this meta-analysis found no significant differences in such complications between the two groups, although an incidence of changes in adjacent-level range of motion were significantly higher in the ACDF group than in the CDA group [29].

CDA produced significantly greater improvement in neck pain VAS than ACDF, while improvements in other pain scores, the NDI, and JOA scores appear to be comparable in this meta-analysis. In one of the studies considered, focused on treating two-level disease, significant improvements were observed in the VAS neck, VAS arm, NDI, and JOA scores compared to the baseline values in both groups. However, there were distinct differences between the two treatment groups. In the CDA group, there was a significant increase in the range of motion at the index levels. On the other hand, the ACDF group showed a significant limitation in the range of motion at the index levels [28]. The authors of that study suggested that motion preservation after CDA reduced stress on the adjacent segments that were non-operated, ultimately decreasing disc degeneration at those levels. Given the maintenance of cervical kinematics associated with CDA, it remains an efficacious alternative to ACDF. However, despite the findings of the present updated meta-analysis, along with those of ongoing studies on CDA and ACDF, two-level CDA still warrants large-scale randomized clinical trials in order to confirm its safety and efficacy.

Lastly, regarding CDA, surgeons may be interested in the risk heterotopic ossification (HO) in the long run [36]. The long-term study by Gornet et al. [26] documented that the incidence of severe HO (grade III or IV) did not significantly increase after 7 years. However, this points to a limitation in our meta-analysis, given that the follow-up durations in other included studies were not long enough to adequately report on HO. Consequently, there is a need for future research with extended follow-up periods to conduct a more detailed analysis of HO following CDA.

A research team based in Toronto, Canada, conducted a comprehensive study summarizing the latest trends in both basic and clinical research within our field. This includes advancements in imaging, clinical diagnostic tools, molecular genetics, surgical techniques, and reparative/regenerative strategies [37]. Our present meta-analysis reflects the rapid progress within this field and addresses a critical research question posed by the Toronto team. It significantly contributes insights into customized surgical strategies for patients with mild degenerative cervical myelopathy and radiculopathy.

### Strengths and Limitations

The present meta-analysis reviewed the most recent studies—most were conducted within five years—and included up-to-date evidence on the postoperative outcomes of CDA versus ACDF performed for two-level cervical DDD. Despite these strengths, our study encountered several limitations. In this meta-analysis, out of eight studies included, only two were RCTs, and the study numbers for certain outcomes were small, which could potentially lower the overall evidence level of this meta-analysis. Although we assessed complication rates, the included studies did not provide sufficient data for a comparison on implant-related and surgery-related complications between CDA and ACDF. Crucially, while we evaluated the rates of secondary surgery, it remains uncertain whether these surgeries were necessitated by ASD, implant failure, or complications arising from the initial surgeries due to limitations in the available data. Intraoperative parameters such as operative times, surgeons’ experience, and medications prescribed were not considered in the present analysis, because these factors were not provided consistently for all patients and were not adjusted in the included studies. The range of follow-up duration was fairly wide, but no heterogeneity was detected across the studies regarding the primary outcomes assessed. More research efforts and prospective clinical studies are required to confirm the results of the present study and broaden the understanding of differences between CDA and ACDF in treating patients with two-level cervical DDD.

## 5. Conclusions

The postoperative outcomes of CDA show a higher rate of surgical success and a lower rate of secondary surgeries than ACDF, while providing a greater reduction in pain and fewer postoperative complications than ACDF for treating patients with two-level cervical DDD.

## Figures and Tables

**Table 1:** Characteristics of the selected studies and included patients.

First Author	Year of Publication	Study Design	Number of Patients	Mean Age (y)	Male (n, %)	Length of Follow-Up
Bakare [23]	2022	Retrospective	CDA: 32 ACDF: 50	CDA: 46.7 ACDF: 58.0	CDA: 53% ACDF: 46%	1 year
Sharma [24]	2022	Retrospective	CDA: 27 ACDF: 22	TDR: 43.4 ACDF: 53.4	TDR: 74% ACDF: 63%	3–4 years
Gao [25]	2019	Retrospective	CDA: 24 ACDF: 36	CDA: 54.7 ACDF: 58.6	CDA: 63% ACDF: 61%	5 years
Gornet [26]	2019	RCT	CDA: 209 ACDF: 188	CDA: 47.1 ACDF: 47.6	CDA: 44% ACDF: N/A	10 years
Radcliff [27]	2017	RCT	CDA: 225 ACDF: 105	TDR: 45.3 ACDF: 46.2	TDR: 50% ACDF: 43%	7 years
Fay [28]	2014	Retrospective	CDA: 37 ACDF: 40	CDA: 52.1 ACDF: 63.0	CDA: 76% ACDF: 65%	2 years
Hou [29]	2014	Prospective	CDA: 32 ACDF: 88	CDA: 46.9 ACDF: 51.2	CDA: 63% ACDF: 43%	22.5 months
Kim [30]	2009	Prospective	CDR: 12 ACDF: 28	CDR: 46.9 ACDF: 52.7	CDR: 67% ACDF: 61%	13–37 months

RCT, randomized controlled trial; CDA, cervical disc arthroplasty; ACDF, anterior cervical discectomy and fusion.

**Table 2:** Sensitivity analysis of study outcomes.

	Statistics with Study Removed				
	Points	Lower Limit	Upper Limit	Z-Value	*p*-Value
Overall success rate					
Gornet 2019 [26]	2.984	1.839	4.842	4.428	0.000
Radcliff 2017 [27]	2.493	1.588	3.914	3.968	0.000
Secondary surgery rate					
Bakare 2022 [23]	0.251	0.165	0.383	−6.420	0.000
Gornet 2019 [26]	0.251	0.121	0.521	−3.707	0.000
Radcliff 2017 [27]	0.260	0.162	0.416	−5.620	0.000
NDI					
Bakare 2022 [23]	−0.059	−0.438	0.320	−0.304	0.761
Sharma 2022 [24]	0.251	−0.286	0.788	0.916	0.360
Gao 2019 [25]	0.310	−0.178	0.799	1.245	0.213
Radcliff 2017 [27]	0.137	−0.520	0.793	0.408	0.683
Fay 2014 [28]	0.199	−0.377	0.775	0.676	0.499
Hou 2014 [29]	0.224	−0.348	0.796	0.768	0.443
Kim 2009 [30]	0.074	−0.459	0.607	0.273	0.785
Neck pain VAS					
Bakare 2022 [23]	0.209	0.019	0.399	2.154	0.031
Gao 2019 [25]	0.274	0.107	0.441	3.220	0.001
Radcliff 2017 [27]	0.251	0.005	0.497	1.998	0.046
Fay 2014 [28]	0.266	0.082	0.451	2.827	0.005
Hou 2014 [29]	0.187	0.015	0.359	2.134	0.033
Kim 2009 [30]	0.226	0.034	0.418	2.303	0.021
Arm pain VAS					
Bakare 2022 [23]	−0.114	−0.310	0.082	−1.143	0.253
Sharma 2022 [24]	−1.385	−3.186	0.416	−1.508	0.132
Radcliff 2017 [27]	−1.548	−3.726	0.630	−1.393	0.164
Fay 2014 [28]	−1.549	−3.573	0.476	−1.499	0.134
JOA score					
Bakare 2022 [23]	−0.490	−1.342	0.362	−1.128	0.259
Gao 2019 [25]	2.217	−2.291	6.724	0.964	0.335
Fay 2014 [28]	1.786	−3.574	7.147	0.653	0.514
Complication rate					
Bakare 2022 [23]	0.558	0.330	0.943	−2.179	0.029
Gornet 2019 [26]	0.609	0.321	1.154	−1.520	0.129
Radcliff 2017 [27]	0.526	0.279	0.991	−1.987	0.047
Hou 2014 [29]	0.504	0.272	0.933	−2.181	0.029

## Data Availability

Data are available upon reasonable request from the corresponding author.

## Supplementary Materials

The following supporting information can be downloaded at https://www.mdpi.com/article/10.3390/jcm13113203/s1: Figure S1: (A) Risk of bias summary of RCTs, (B) Risk of bias graph of RCTs; Figure S2: (A) Risk of bias summary of non-RCTs; (B) Risk of bias graph of non-RCTs.
