# Supplementary material for: Cervical Disc Arthroplasty (CDA) versus Anterior Cervical Discectomy and Fusion (ACDF) for Two-Level Cervical Disc Degenerative Disease: An Updated Systematic Review and Meta-Analysis
Source: J Clin Med. 2024 May 29;13(11):3203. doi: 10.3390/jcm13113203 (PMC11173267; doi:10.3390/jcm13113203)

Supplemental Figure S1A. Risk of bias summary of RCTs.

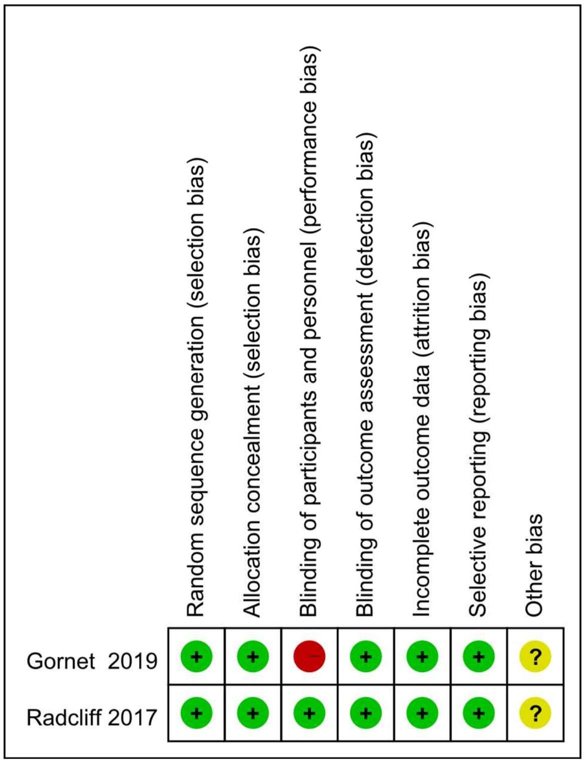

Supplemental Figure S1B. Risk of bias graph of RCTs.

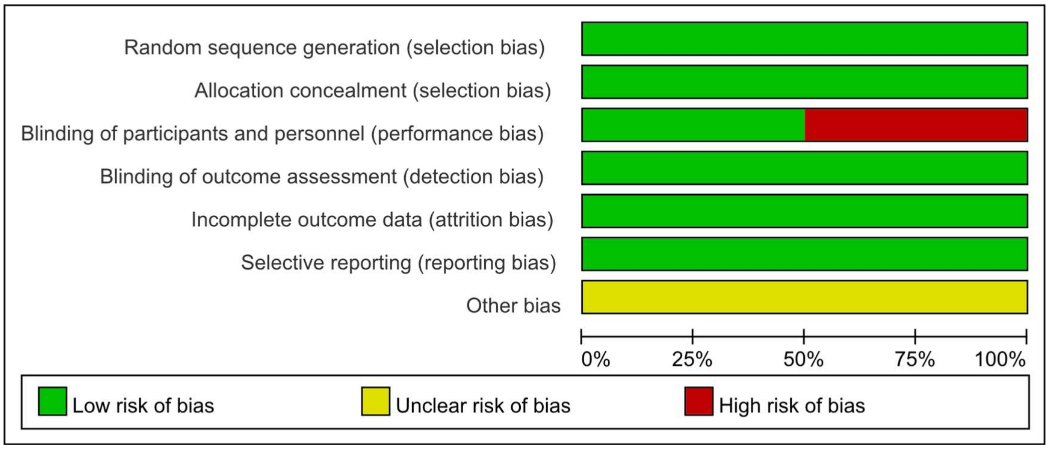

Supplementary Figure S2A. Risk of bias summary of non-RCTs

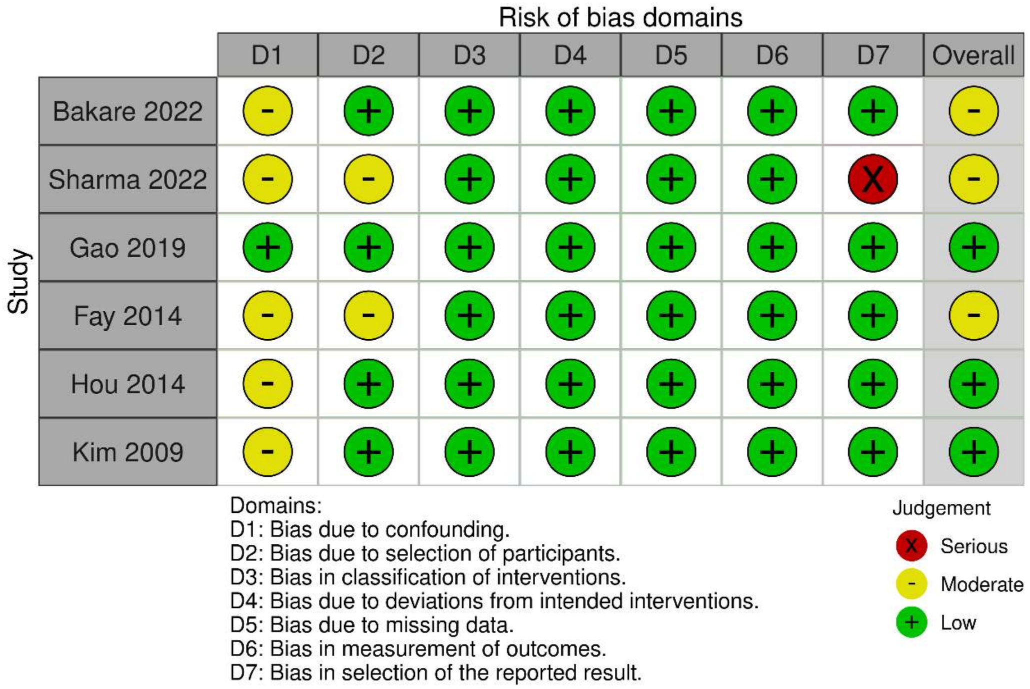

Supplementary Figure S2B. Risk of bias graph of non-RCTs

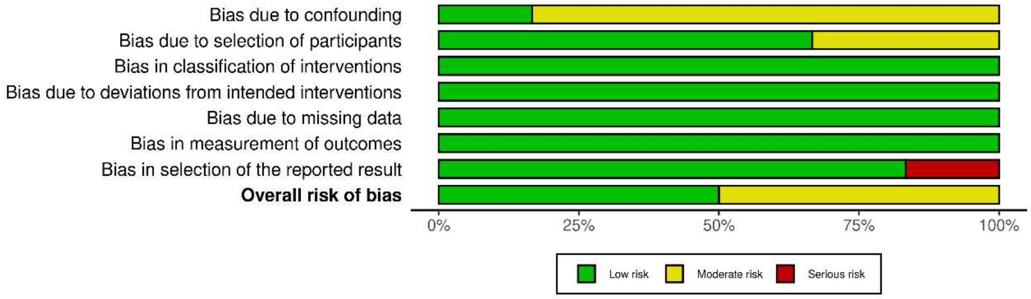

Supplement: Supplementary file 1 [file jcm-13-03203-s001.zip › jcm-2873368-supplementary.pdf]
